# Supplementary material for: Comparative transcriptome analyses in contrasting onion (Allium cepa L.) genotypes for drought stress
Source: PLoS One. 2020 Aug 11;15(8):e0237457. doi: 10.1371/journal.pone.0237457 (PMC7418993; doi:10.1371/journal.pone.0237457)
Supplement: S1 Table — (DOCX) [file pone.0237457.s001.docx]

**S1 Table: The interProScan analysis**

| **Database** | **Description (as per InterProScan Documentation)** | **1627D** | **1656D** |
| --- | --- | --- | --- |
| Phobius | A combined transmembrane topology and signal peptide predictor | 432241 | 372122 |
| TMHMM | Prediction of transmembrane helices in proteins | 125958 | 107372 |
| MobiDBLite | Prediction of disordered domains Regions in Proteins | 25208 | 23290 |
| PANTHER | The PANTHER (Protein ANalysis THrough Evolutionary Relationships) Classification System is a unique resource that classifies genes by their functions, using published scientific experimental evidence and evolutionary relationships to predict function even in the absence of direct experimental evidence. | 13369 | 13051 |
| Coils | Prediction of Coiled Coil Regions in Proteins | 8224 | 7314 |
| Pfam | A large collection of protein families, each represented by multiple sequence alignments and hidden Markov models (HMMs) | 7355 | 7335 |
| Gene3D | Structural assignment for whole genes and genomes using the CATH domain structure database | 6180 | 6099 |
| SUPERFAMILY | SUPERFAMILY is a database of structural and functional annotation for all proteins and genomes. | 5080 | 4995 |
| SignalP_EUK | SignalP (organism type eukaryotes) predicts the presence and location of signal peptide cleavage sites in amino acid sequences for eukaryotes. | 5050 | 4627 |
| ProSiteProfiles | PROSITE consists of documentation entries describing protein domains, families and functional sites as well as associated patterns and profiles to identify them | 3908 | 3972 |
| PRINTS | A fingerprint is a group of conserved motifs used to characterise a protein family | 2823 | 2731 |
| SMART | SMART allows the identification and analysis of domain architectures based on Hidden Markov Models or HMMs | 2691 | 2568 |
| CDD | Prediction of CDD domains in Proteins | 1785 | 1730 |
