# Supplementary figures and images for: Comparative transcriptome analyses in contrasting onion (Allium cepa L.) genotypes for drought stress
Source: PLoS One. 2020 Aug 11;15(8):e0237457. doi: 10.1371/journal.pone.0237457 (PMC7418993; doi:10.1371/journal.pone.0237457)

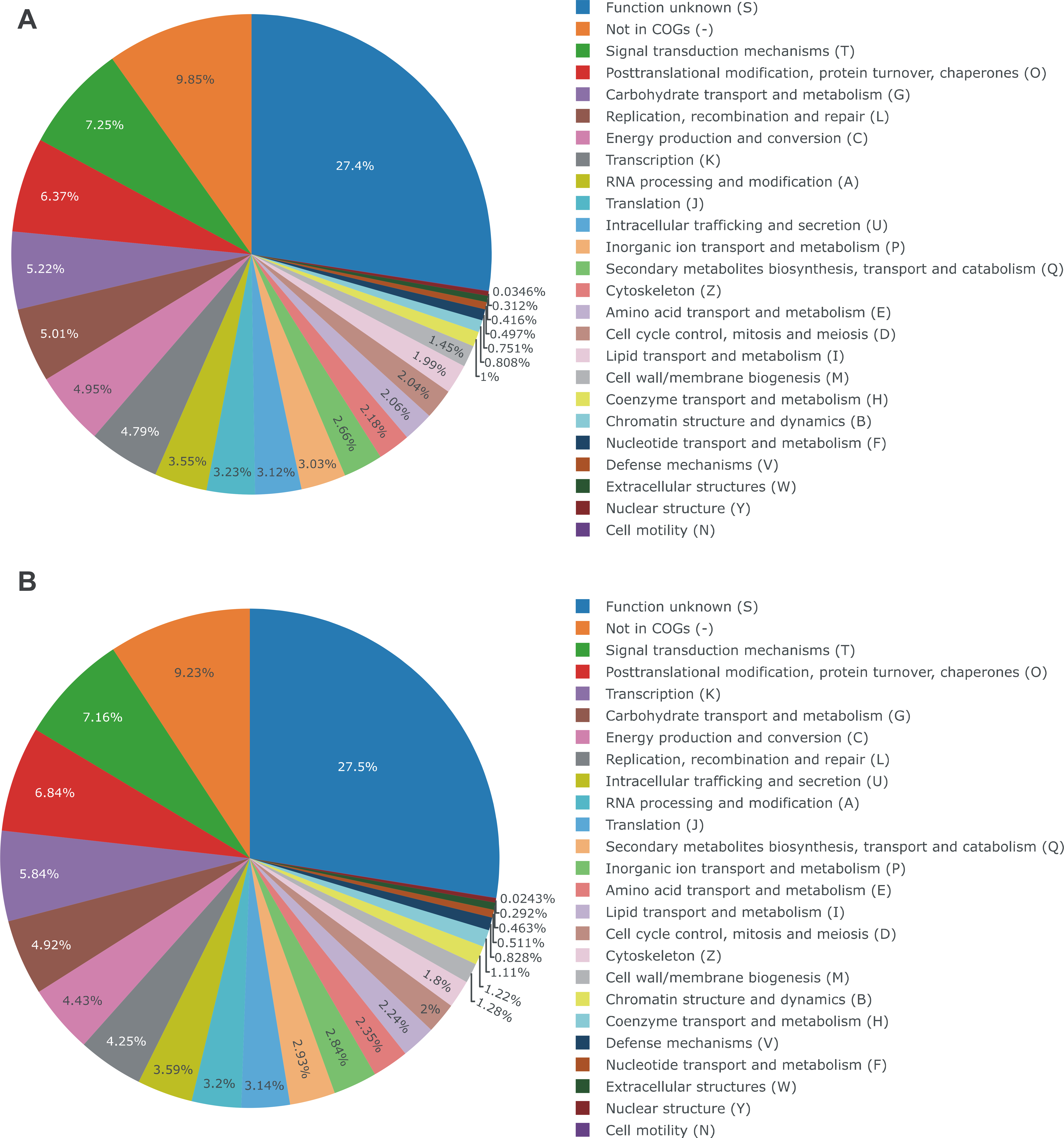

Supplement: S1 Fig — (TIF) [file pone.0237457.s002.tif]
