# Supplementary material for: Comparative transcriptome analyses in contrasting onion (Allium cepa L.) genotypes for drought stress
Source: PLoS One. 2020 Aug 11;15(8):e0237457. doi: 10.1371/journal.pone.0237457 (PMC7418993; doi:10.1371/journal.pone.0237457)
Supplement: Attachment — Submitted filename: PONE-D-20-06651_Reviewers comments.docx [file pone.0237457.s008.docx]

Though RNA-Seq based works are very common now-a-days for investigating role, discovery of candidate genes and markers and differential gene expression profiling, yet the work entitled “Comparative transcriptome analyses in contrasting onion (*Allium cepa* L.) genotypes for drought stress” is important in two respects, one the crop is commercially important and second, drought stress which causes huge yield loss. I commend the authors for this study, and would like some clarity on following points:

**Comment 1:**

“Authors performed de novo DGE analyses using aligned reads of drought-tolerant (1656C vs 1656D) and drought-sensitive (1627C vs 1627D) onion cultivars”.

I believe, the comparative analysis should have been made also between 1627D vs 1656D and 1627C vs 1656C. This could be important in better understanding the drought stress in tolerant and susceptible genotype.

**Comment 2:**

Why not genes were selected based on their high fold change value (negative and positive) and drought-related instead of random selection just for validation of RNA-Seq identified DGEs. Also the gene expression analysis at 6, 12, 18, 24 days should have been performed to correlate with biochemical and physiological data related to drought stress.

**Comment 3:**

The study comes out with a long list of drought stress-related such as genes encoding transcription factors, cytochrome P450, membrane transporters, flavonoids, and carbohydrate metabolism, etc. which showed differential expression behavior in tolerant and susceptible genotypes, it would not be much helpful in understanding the key role players. Can authors enlist few candidate genes for drought stress in onion based on present study?

**Comment 4:**

“Total phenol content that directly linked with the onion pungency and found to be elevated in response to drought stress”. Why none of the genes related to phenylpropanoid/Flavonoid pathway were included qRT-PCR.

**Comment 5:**

Discussion lacks on up-regulated genes in tolerant genotype like WAT 1 related protein, NINJA family AFP1, Methyl malonyl co-A epimerase and 21KDa Protein.

**Comment 6:**

Some figures labels needs clarity such as Figure 3 and Figure 5, the labels are difficult to read.
