# Supplementary material for: Comparative transcriptome analyses in contrasting onion (Allium cepa L.) genotypes for drought stress
Source: PLoS One. 2020 Aug 11;15(8):e0237457. doi: 10.1371/journal.pone.0237457 (PMC7418993; doi:10.1371/journal.pone.0237457)
Supplement: Attachment — Submitted filename: Response to Reviewers.doc [file pone.0237457.s009.doc]

**5. Review comments to the Author**

Please use the space provided to explain your answers to the questions above. You may also include additional comments for the author, including concerns about dual publication, research ethics, or publication ethics. (Please upload your review as an attachment if it exceeds 20,000 characters)

***Reviewer #1:*** Though RNA-Seq based works are very common now-a-days for investigating role, discovery of candidate genes and markers and differential gene expression profiling, yet the work entitled “Comparative transcriptome analyses in contrasting onion (*Allium cepa* L.) genotypes for drought stress” is important in two respects, one the crop is commercially important and second, drought stress which causes huge yield loss. I commend the authors for this study, and would like some clarity on following points:

**Comment 1:**

“Authors performed de novo DGE analyses using aligned reads of drought-tolerant (1656C vs 1656D) and drought-sensitive (1627C vs 1627D) onion cultivars”. I believe, the comparative analysis should have been made also between 1627D vs 1656D and 1627C vs 1656C. This could be important in better understanding the drought stress in tolerant and susceptible genotype.

**Response:** We do agree with this comment. We would be happy to perform additional analysis suggested by the reviewer. But, due to Covid-19 pandemic, our research institute and Pune University are not being fully functional. We are in hotspot zone, our access to labs have been restricted. The present lockdown has been extended till 31^st^ July, 2020, since positive cases are going up substantially, lockdown is most likely extended beyond 31^st^ July. Therefore, we could not able to perform the suggested additional analysis. However, we feel that the analyses in the submitted manuscript are adequate enough to support the proposed study. Further, we accept the suggestion and we are planning to publish the detailed comparative analysis of 1627D vs. 1656D and 1627C vs. 1656C as a sequel to the present manuscript as and when things gets normal.

**Comment 2:**

Why not genes were selected based on their high fold change value (negative and positive) and drought-related instead of random selection just for validation of RNA-Seq identified DGEs. Also the gene expression analysis at 6, 12, 18, 24 days should have been performed to correlate with biochemical and physiological data related to drought stress.

**Response:** The purpose of the qRT-PCR in the present study was only to validate the transcriptomics data**.** Most of the transcripts we selected for qRT-analysis were directly linked with the drought stress. We already have performed biochemical analyses of drought related markers at different time points (6, 12, 18, 24 days). Many of the genes showed high fold change values were linked to these markers. The additional gene expression analysis would not add any additional information to the manuscript. The suggested analysis would have strengthened the validation only that we have adequately performed in the manuscript.

**Comment 3:**

The study comes out with a long list of drought stress-related such as genes encoding transcription factors, cytochrome P450, membrane transporters, flavonoids, and carbohydrate metabolism, etc. which showed differential expression behaviour in tolerant and susceptible genotypes, it would not be much helpful in understanding the key role players. Can authors enlist few candidate genes for drought stress in onion based on present study?

**Response:** Yes, we have listed few genes which showed multi-fold upregulation under drought stress in result section and their supporting discussion was incorporated in revised MS.

**Comment 4:**

“Total phenol content that directly linked with the onion pungency and found to be elevated in response to drought stress”. Why none of the genes related to phenylpropanoid/Flavonoid pathway were included qRT-PCR.

**Response:** Thanks for the suggestion; we already performed total phenol analysis that is strongly linked with the pungency. The purpose of the qRT-PCR in the present study was only to validate the transcriptomics data. Therefore, we randomly selected genes for qRT-PCR to validate the RNAseq data without targeting any pathway. However, we will certainly target the Phenylpropanoid/Flavonoid pathways for further probing into the drought mechanisms in our future studies.

**Comment 5:**

Discussion lacks on up-regulated genes in tolerant genotype like WAT 1 related protein, NINJA family AFP1, Methyl malonyl co- A epimerase and 21KDa Protein.

**Response:** As per suggestion, we have added discussion on suggested genes in revised MS.

**Comment 6:**

Some figures labels needs clarity such as Figure 3 and Figure 5, the labels are difficult to read.

**Response:** Figure 3 and 5 are changed in revised MS.

***Reviewer #2:***

Introduction about onion is very shallow. Provide the data of onion production and loss occurs due to other stress and then mention the yield loss due to drought.

**Response**: As per suggestion of reviewer, we have added data in revised MS.

Introduction line no. 70: mention the name of the genotypes.

**Response:** Names of genotypes were mentioned in revised MS

Introduction line no. 75-80 is the part of the result and do not need to describe here

**Response:** Suggested part is deleted in revised MS

MM: 89: 1656 and 1627 are the cv nos? specify clearly.

**Response:** These are accession number of genotypes under study. It is specified in revised MS.

MM: irrigated at 100% field capacity until they reached the 5-6 leaf stage, how come it is mentioned as field capacity, though plants are in pots.

**Response:** These plants were watered regularly as per the crop requirement so that they do not feel water stress

L188: mention the name of selected genes, and site the table for the primers here.

**Response:** Changes made as per suggestion in revised MS.

Fig1 A: change sample as genotypes and treatments.

**Response:** Changes made in figure as per suggestion

Morphological data should be added in main figure, shift from supplementary to main text.

**Response:** Morphological data is added in main manuscript as Figure 2.

L 200: significantly maintained the number of leaves, is it higher then edit the sentence as

Significantly maintained higher number of leaves

**Response**: Sentence modified as per suggestion

For membrane damage, authors are advised to include MDA data.

**Response:** Drought stress imposed at various stages of crop growth resulted in an increase of oxidative stress that causes considerable cellular membrane damage. The extent of damage to membranes was reflected by two main indicators i.e. cellular membrane stability index (MSI%) and lipid peroxidation (Accumulation of MDA). Both these parameter are most effective approach for quantifying the level of plant water stress. In the present work we quantify the cellular membrane stability index instead of lipid peroxidation as a consistent and good parameter indicating the cellular membrane damage in response to water stress. The data for MSI was included in Manuscript.

L 208, 209: incomplete sentence, please edit it: The observation recorded for leaf chlorophyll content was also in the same line 209 differing among the genotypes subjected to drought stress.

**Response:** Sentence is reframed as reviewer’s suggestion

L 212: Total phenol content that directly linked with the onion pungency and found to be elevated in response to drought stress: and should be was

**Response:** Sentence modified as per suggestion

L258: needs to be place before and after respectively.

**Response**: Changes made in revised MS

Fig 3A-F, Fig. 5: font size is too small and is not readable.

**Response:** Figures are changed in revised submission

L 305-353: data mentioned in the result section should be properly checked as at some point the fold expression has not been mentioned. Authors should uniformly mention the fold expression, where it upregulated or down regulated

**Response:** Fold changes were mentioned in revised MS**.**

L 354-359: The real time expression in the result section is written as MM part and result is missing. Pl write the result part properly. Move this write up in MM.

**Response:** Result part is modified in revised MS as per reviewer’s suggestion

Prediction of SSR data does not fit relevant.

**Response:** Changes made in revised MS

The authors are advised to correct the MS thoroughly.

**Response:** All advised corrections are made in revised MS
